# Adverse events during intravenous fosfomycin therapy in a real-life scenario. Risk factors and the potential role of therapeutic drug monitoring

**DOI:** 10.1186/s12879-024-09541-4

**Published:** 2024-06-28

**Authors:** Simona Biscarini, Davide Mangioni, Chiara Bobbio, Ludovica Mela, Laura Alagna, Sara Baldelli, Francesco Blasi, Ciro Canetta, Ferruccio Ceriotti, Andrea Gori, Giacomo Grasselli, Bianca Mariani, Antonio Muscatello, Dario Cattaneo, Alessandra Bandera

**Affiliations:** 1 Infectious Diseases Unit, IRCCS Ca’ Granda Ospedale Maggiore Policlinico Foundation, Via Francesco Sforza 35, Milan, 20122 Italy; 2 Pharmacology Unit, Clinical Chemistry Laboratory, ASST Spedali Civili di Brescia, Brescia, Italy; 3 Respiratory Unit and Adult Cystic Fibrosis Center, Fondazione IRCCS Ca’ Granda Ospedale Maggiore, Policlinico, Milan, Italy; 4 Department of Pathophysiology and Transplantation, University of Milan, Milan, Italy; 5 Acute Medical Unit, Fondazione IRCCS Ca’ Granda Ospedale Maggiore Policlinico, Milan, Italy; 6 Clinical Laboratory, Fondazione IRCCS Ca’ Granda Ospedale Maggiore Policlinico, Milan, Italy; 7 Department of Infectious Diseases, ASST-Fatebenefratelli Sacco University Hospital, Milan, Italy; 8 Department of Anaesthesia, Critical Care and Emergency, Fondazione IRCCS Ca’ Granda Ospedale Maggiore Policlinico, Milan, Italy

**Keywords:** Intravenous fosfomycin disodium, Adverse events, Toxicity, Multidrug resistance, Antimicrobial resistance, TDM

## Abstract

**Background:**

Intravenous fosfomycin (IVFOF) is gaining interest in severe infections. Its use may be limited by adverse events (AEs). Little experience exists on IVFOF therapeutic drug monitoring (TDM) in real-life setting.

**Patients and methods:**

Retrospective study of patients receiving IVFOF for > 48 h at Policlinico Hospital (Milan, Italy) from 01/01/2019 to 01/01/2023. AEs associated to IVFOF graded CTCAE ≥ II were considered. Demographic and clinical risk factors for IVFOF-related AEs were analysed with simple and multivariable regression models. The determination of IVFOF TDM was made by a rapid ultraperformance liquid chromatography mass spectrometry method (LC-MS/MS) on plasma samples. The performance of TDM (trough levels (Cmin) in intermittent infusion, steady state levels (Css) in continuous infusion) in predicting AEs ≤ 5 days after its assessment was evaluated.

**Results:**

Two hundred and twenty-four patients were included. At IVFOF initiation, 81/224 (36.2%) patients were in ICU and 35/224 (15.7%) had septic shock. The most frequent infection site was the low respiratory tract (124/224, 55.4%). Ninety-five patients (42.4%) experienced ≥ 1AEs, with median time of 4.0 (2.0–7.0) days from IVFOF initiation. Hypernatremia was the most frequent AE (53/224, 23.7%). Therapy discontinuation due to AEs occurred in 38/224 (17.0%). ICU setting, low respiratory tract infections and septic shock resulted associated with AEs (RR_adjusted_ 1.59 (95%CI:1.09–2.31), 1.46 (95%CI:1.03–2.07) and 1.73 (95%CI:1.27–2.37), respectively), while IVFOF daily dose did not. Of the 68 patients undergone IVFOF TDM, TDM values predicted overall AEs and hypernatremia with AUROC of 0.65 (95%CI:0.44–0.86) and 0.91 (95%CI:0.79-1.0) respectively for Cmin, 0.67 (95%CI:0.39–0.95) and 0.76 (95%CI:0.52-1.0) respectively for Css.

**Conclusions:**

We provided real world data on the use of IVFOF-based regimens and associated AEs. IVFOF TDM deserves further research as it may represent a valid tool to predict AEs.

**Key points:**

Real world data on intravenous fosfomycin for severe bacterial infections. AEs occurred in over 40% (therapy discontinuation in 17%) and were related to baseline clinical severity but not to fosfomycin dose. TDM showed promising results in predicting AEs.

**Supplementary Information:**

The online version contains supplementary material available at 10.1186/s12879-024-09541-4.

## Background

Intravenous fosfomycin (IVFOF) has recently gained interest for the treatment of severe infections, particularly when caused by multidrug-resistant organisms (MDROs) [1]. Its unique mechanism of action provides activity against both gram-positive and gram-negative bacteria and prevents cross-resistance to other class of antibiotics [2]. 

IVFOF is generally prescribed in combination therapy, to avoid the occurrence of resistance and take advantage of its synergism with several other antimicrobials. [3, 4] Recently, it has also showed good results as monotherapy for the treatment of complicated urinary tract infections caused by *Escherichia coli* and other *Enterobacterales*. [5–7]

The use of IVFOF may however be limited by significant adverse events (AEs), specifically electrolyte disorders (hypernatremia and hypokalaemia), gastrointestinal intolerance or cardiac failure due to sodium overload [8, 9]. Furthermore, there is a great variability in IVFOF dosage (12 to 24 g per day, administered 2 to 4 times daily or as a continuous infusion), depending on renal function and source and severity of the infection, with potential risk of under-/over-dosing especially in critically ill patients. [10–12]

Therapeutic drug monitoring (TDM) is proved to be useful for antibiotic dose adjustments for maintaining effective and safe drug concentrations as well as lowering the risk of resistance development. [13] TDM of glycopeptides and aminoglycosides is routinely performed, while TDM of β-lactams is recommended in critically ill patients with altered pharmacokinetic/pharmacodynamic (PK/PD) parameters. [14–16] To date, IVFOF TDM is not routinely performed in most centers due to the lack of commercial kits for drug quantification and the absence of established cut-offs for drug safety and/or efficacy. However, observations from single case reports or small cases series have provided preliminary evidence on the role of TDM in optimizing IVFOF administration. [17–21]

Here we report our real-life experience of IVFOF-based antibiotic therapies in a large cohort of patients with severe bacterial infections, with a focus on drug-related AEs and factors associated with their development.

## Patients and methods

### Study design and setting

Real-life, single centre retrospective cohort study of patients treated with IVFOF at Foundation IRCCS Ca’ Granda Ospedale Maggiore Policlinico (Milan, Italy) from January 1, 2019 to January 1, 2023. InfectoFos^®^ (InfectoPharm s.r.l., Milan, Italy) preparation for intravenous use is employed in our Institution; IVFOF dosage was chosen based on source of infection, microbial isolates and renal function according to technical data sheet. Each gram contains 14 mEq (320 mg) of sodium. [22] As per clinical practice in our Hospital, IVFOF TDM was not standardised but requested on a case-by-case basis by the infectious diseases (ID) consultant in charge of the patient management. Samples are usually collected into EDTA plasma sampling tubes ≥ 48 h from IVFOF start, centrifuged and sent to the laboratory of the Unit of Clinical Pharmacology ASST Fatebenefratelli Sacco (Milan, Italy) on ice and frozen at -20 °C until analysis. The laboratory processes IVFOF TDM samples twice weekly using a rapid ultraperformance liquid chromatography mass spectrometry method as previously described. [23] In the absence of established cut off and clear IVFOF PK/PD parameters of microbiological eradication and clinical efficacy, TDM values were managed by the ID consultant in collaboration with the Clinical Pharmacologist. We considered IVFOF with a time-dependent killing activity (PK/PD index: T_> MIC_) based on previous studies [12, 24–26] and the microbiological characteristics of our cohort, with the majority of isolates represented by *Pseudomonas aeruginosa*and*Staphylococcus aureus*(Table S1). The value of 100% T_> MIC_ (or ECOFF, if MIC not available) of the isolated pathogen was conservatively and arbitrarily chosen as the time-dependent killing activity index.

### Study participants and data collection

All consecutive patients treated with IVFOF were considered for inclusion. Exclusion criteria was length of IVFOF treatment ≤ 48 h (discontinuation not due to AEs) and lack of data on the primary outcome (no information on AEs).

Demographic, clinical, laboratory and outcome data were collected from clinical records. Microbiological and therapeutic data were collected from dedicated hospital-databases.

Bacterial isolates were defined as MDROs when non-susceptible to at least one agent in three or more antimicrobial categories or when harbouring specific antibiotic resistance mechanisms (e.g., methicillin-resistant *Staphylococcus aureus*; vancomycin-resistant *Enterococcus faecium*; extended spectrum beta-lactamase (ESBL)- or carbapenemase-producing *Enterobacterales*). [27]

AEs were registered if: (i) reported by IVFOF technical data sheet [28], (ii) had severity grade II or higher according to Common Terminology Criteria for Adverse Events (CTCAE) Version 5.0 [29] and (iii) occurred from the first day of IVFOF administration until 10 days after its discontinuation. Specifically, the following AEs were registered: diarrhoea, if ≥4 stools/day; nausea, when resulting in reduced oral intake; hypernatremia, if evidence of sodium ≥150 mmol/L; hypokalemia, if evidence of potassium < 3 mmol/L with symptoms associated; hypertransaminasemia if evidence of alanine transaminase (ALT) > 3.0 x upper normal limit or > 3.0 x baseline if baseline was abnormal; any registered cardiac event (e.g., development of arrhythmias, QT prolongation, cardiac arrest).

TDM values were categorized as trough concentration (minimum concentration, Cmin) when IVFOF was administered as intermittent infusion (II) and the sample was collected 30 min before the next dose administration, or as steady-state concentration (Css) in case of continuous infusion (CI). For patients treated with multiple courses of IVFOF or managed with repeated TDM, only first IVFOF employment and first TDM assessment were considered.

The primary outcome was to investigate epidemiological and clinical factors associated with the development of moderate or severe IVFOF-related AEs. Secondary outcome was to evaluate whether IVFOF TDM could predict the development of AEs within 5 days from its assessment (AEs_≤ 5days_). The 5-day interval was set to include events occurred before treatment changes based on TDM.

Anonymized data were abstracted on standardized data collection forms in the web platform REDCap (Reaserch Electronic Data Capture). [30, 31]

The study was registered by the Milan Area 2 Ethical Committee (#664_2022bis) and was conducted in accordance with standards of the Helsinki Declaration. Informed consent for pseudonymized data processing for future research purposes was provided by all patients at the time hospital admission, as routine procedure. Specific written informed consent was waived because of the retrospective nature of the analysis.

### Statistical analysis

Continuous variables were presented as medians and first and third quartiles (Q1-Q3), categorical variables were reported as frequencies and proportions. Group comparisons were conducted using appropriate statistical tests depending on the variable distribution, including the Mann-Whitney test for continuous variables and the Chi-squared test or Fisher’s exact test for categorical variables.

For the analysis of factors associated with the occurrence of IVFOF-related AEs, log-binomial regression models were used, or Poisson regression models with robust error variance when the log-binomial model failed to converge. Risk ratios (RRs) were estimated, along with their corresponding 95% CIs. [32] In all the multivariable regression models, factors were entered into the adjusted model on the basis of their univariate relation to outcome (*p* < 0.20) along with possible confounders. All factors were biologically plausible with a sound scientific rationale. However, if the Pearson or Spearman correlation coefficient (according to variables distribution), was > 0.20, the variable with the lower p-value was retained in the model (for example, when septic shock with vasopressors was considered, IVFOF treatment started in ICU was excluded). Confounders included in multivariable models are reported in footnotes.

To evaluate the performance of TDM values in predicting at least one AEs_≤ 5days_ or hypernatremia_≤ 5days_, receiver operating characteristic (ROC) curves were constructed and their corresponding areas under the curve (AUC) were evaluated in both groups of IVFOF CI and II.

Statistical analyses were performed using SAS 9.4 software (Cary, NC, USA). For all tests, a two-tailed significance level was considered.

## Results

### Study population

**Fig. 1:**
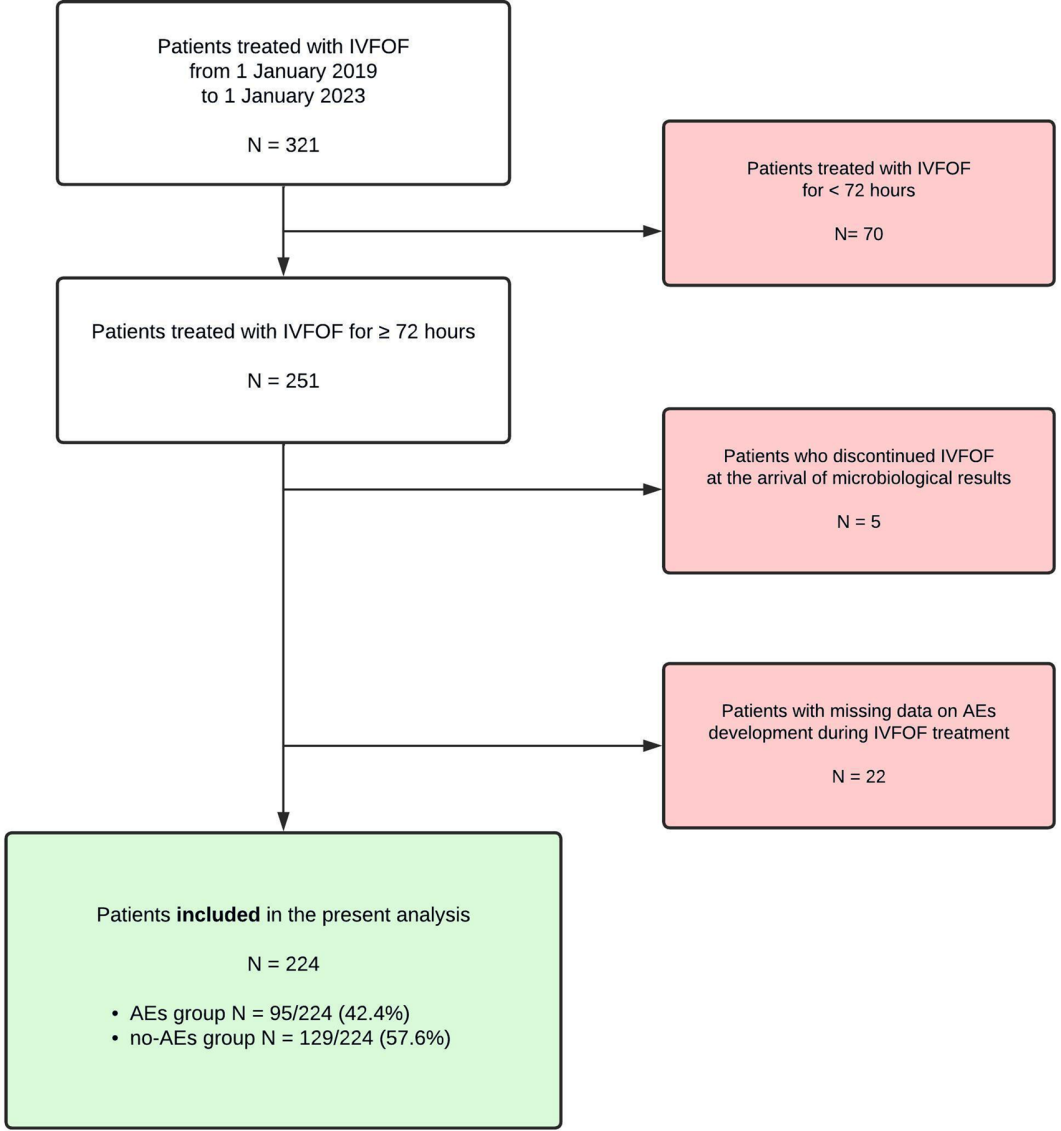
Flowchart of the study population

Three-hundreds and twenty-one patients were considered. Of them, 70 were excluded from the analysis because treated for ≤ 48 h (surgical prophylaxis, early interruption due to death or other causes unrelated to treatment), 5 because IVFOF was started as empiric therapy and discontinued once microbial results were available and 22 patients for missing data related to the primary outcome (Fig. 1).

Demographic and clinical characteristics of the study cohort are reported in Table 1, overall and for patients who did and did not develop IVFOF-related AEs.

**Table 1:** Demographic and clinical characteristics of the overall study population. Comparison between patients who developed adverse events (AEs) or not (no-AEs) related to the use of IVFOF

	Study population (*N* = 224)	AEs (*N* = 95)	no-AEs (*N* = 129)	*p*-value
Demographics				
Age, years	63.0 (50.0-71.5)	66.0 (55.0–73.0)	59.0 (46.0–71.0)	0.025
Gender, female	86 (38.4)	36 (37.9)	50 (38.8)	0.895
Ethnicity, Caucasian	201 (89.7)	88 (92.6)	113 (87.6)	0.220
Comorbidities				
At least 1 comorbidity ^a^	146 (65.5)	65 (68.4)	81 (63.3)	0.425
Myocardial infarction	25 (11.2)	13 (13.7)	12 (9.3)	0.303
Chronic pulmonary disease	39 (17.4)	15 (15.8)	24 (18.6)	0.583
Mild or severe liver disease	24 (10.7)	10 (10.5)	14 (10.9)	0.938
Diabetes Mellitus	67 (29.9)	28 (29.5)	39 (30.2)	0.902
Charlson Comorbidity Index				
0	77 (34.4)	30 (31.6)	47 (36.4)	0.329
1	48 (21.4)	21 (22.1)	27 (20.9)	
2	43 (19.2)	24 (25.3)	19 (14.7)	
3	33 (14.7)	12 (12.6)	21 (16.3)	
≥ 4	23 (10.3)	8 (8.4)	15 (11.6)	
**Clinical and laboratory data at IVFOF initiation**				
Patient’s ward				
ICU	81 (36.2)	47 (49.5)	34 (26.4)	**< 0.001**
Non-intensive wards ^b^	143 (63.8)	48 (50.5)	95 (73.6)	
Infection site ^c^				
BSI	72 (32.1)	28 (29.5)	44 (34.1)	0.463
Primary BSI	19 (8.5)	6 (6.3)	13 (10.1)	0.318
Lower respiratory tract infection	124 (55.4)	62 (65.3)	62 (48.1)	**0.011**
Surgical site infection	12 (5.4)	5 (5.3)	7 (5.4)	0.957
Urinary tract infection	13 (5.8)	4 (4.2)	9 (7.0)	0.565
Skin and soft tissue infection	11 (4.9)	4 (4.2)	7 (5.4)	0.763
Cardiovascular infection	17 (7.6)	8 (8.4)	9 (7.0)	0.687
Osteoarticular infection	16 (7.1)	5 (5.3)	11 (8.5)	0.349
Others ^c^	23 (10.3)	7 (7.4)	16 (12.4)	0.220
Septic shock with vasopressors need ^a^	35 (15.7)	24 (25.5)	11 (8.5)	**< 0.001**
eGFR, ml/min/1.73m^2 a, d^	93.5 (61.6-110.8)	84.3 (52.2–107.0)	98.2 (67.4–113.0)	0.016
eGFR, ml/min/1.73m^2^ <= 30 ^a, d^	16 (7.8)	10 (11.6)	6 (5.1)	0.086
Hypernatremia	8 (3.6)	6 (6.3)	2 (1.6)	0.074
**Microbiological data at IVFOF initiation**				
Non-identified pathogen	21 (9.4)	5 (5.3)	16 (12.4)	0.070
Identified pathogen	203 (90.6)	90 (94.7)	113 (87.6)	
Monomicrobial infection	153 (75.4)	62 (68.9)	91 (80.5)	0.056
Polymicrobial infection	50 (24.6)	28 (31.1)	22 (19.5)	
Infection sustained by MDROs	99 (48.3)	41 (45.6)	58 (51.3)	0.414
**Treatment data & AEs**				
IVFOF daily dose, grams	16.6 (12.0–24.0)	18.0 (13.4–24.0)	16.0 (12.0–24.0)	0.234
Days elapsed from pathogens identification to IVFOF start ^e^	3.0 (1.0–7.0)	3.0 (1.0–6.0)	3.0 (1.0–7.0)	0.916
IVFOF mode of administration				
Intermittent	190 (84.8)	74 (77.9)	116 (89.9)	**0.013**
Continuous infusion	34 (15.2)	21 (22.1)	13 (10.1)	
IVFOF TDM performed	68 (30.4)	37 (39.0)	31 (24.0)	**0.016**
Length of IVFOF therapy, days	11.0 (7.0-16.5)	9.0 (6.0–16.0)	13.0 (8.0–18.0)	**0.024**
Reason to IVFOF treatment interruption				
Death	14 (6.3)	6 (6.3)	8 (6.2)	**< 0.001**
Toxicity	38 (17.0)	33 (34.7)	5 (3.9)	
Clinical failure	33 (14.7)	15 (15.8)	18 (14.0)	
Clinical cure	139 (62.1)	41 (43.2)	98 (76.0)	
Adverse events, related to IVFOF				
≥ 1 adverse event	95 (42.4)	95 (100)	-	-
Diarrhoea	20 (8.9)	20 (21.1)	-	-
Nausea	12 (5.4)	12 (12.6)	-	-
Hypernatremia	53 (23.7)	53 (55.8)	-	-
Hypertransaminasemia	12 (5.4)	12 (12.6)	-	-
Hypokalemia	22 (9.8)	22 (23.2)	-	-
Cardiac events ^f^	5 (2.2)	5 (5.3)	-	-
Days elapsed from IVFOF start to first AE	4.0 (2.0–7.0)	4.0 (2.0–7.0)	-	-

Data are presented according the development (or not) of at least one AES. Crude and adjusted RRs and their relative 95% CI are reported

Legend: AE Adverse event, BSI blood stream infection, CTCAE common terminology criteria for adverse events, eGFR estimated glomerular filtration rate, ICU intensive care unit, IVFOF intravenous fosfomycin, KDIGO kidney disease improving global outcomes, MDROs multidrug resistant organisms, TDM therapeutic drug monitoring, VAP ventilator associated pneumonia;

^a^ Sum does not add to the total because of missing values: 1 for at least one comorbidity, 1 for Septic shock with vasopressors need, and 1 for eGFR

^b^ 10/224 (4.5%) patients started IVFOF before ICU admission and 19/224 (8.5%) started IVFOF after ICU stay. 4/95 (4.2%) patients in the AEs group and 6/129 (4.7%) patients in the no-AEs group started IVFOF before ICU admission. 8/95 (8.4%) patients in the AEs group and 11/129 (8.5%) patient in the no-AEs group started IVFOF after ICU stay

^c^ 63 patients (28%) had multiple site infections. 27/95 (28.4%) were in the AEs group and 36/129 (27.9%) were in the no-AEs group

^d^*N* = 204, excluding 18 patients (8 patients in the AE group and 10 in the no-AE group) in renal replacement therapy at the initiation of IVFOF and 1 patient for whom the information was not available

^e^*N* = 203, excluding the 21 infections with non-identified pathogen

^f^ Cardiac events defined as development of arrhythmias, QT prolongation, cardiac arrest

Overall, median age was 63.0 (50.0-71.5) years, 86/224 (38.4%) were female and 4/224 (1.8%) were under 18 years of age. One hundred and forty-six patients (65.5%) had at least one chronic illness, with diabetes mellitus being the most frequent (67/224, 29.9%). The majority of patients (203/224, 90.6%) had a microbiologically defined infection, with MDROs in almost half of them (99/203, 48.3%). The most frequent infection site was represented by the lower respiratory tract (124/224, 55.4%). Bloodstream infections (BSI) were 72/224 (32.1%), with 53 cases secondary to another infectious site and 19/224 (8.5%) primary BSI. At IVFOF initiation, 81/224 (36.2%) patients were admitted in the ICU and 35/224 (15.7%) had septic shock. Median length of hospitalization was 43 (26-75.5) days and overall in-hospital mortality was 60/224 (26.8%), confirming the high clinical severity of the population in analysis. IVFOF was administered at a median daily dose of 16.6 (12.0–24.0) grams, started within 3 (1–7) days from pathogen identification. The majority of patients (190/224, 84.8%) received IVFOF as II, 34/224 (15.2%) as CI. Median length of treatment was 11.0 (7.0-16.5) days. IVFOF was employed as part of combination therapy in all but 4 cases (2 infections sustained by *Enterobacterales* and 2 empirical therapies based on previous *Enterobacterales* infection/colonization). Details on microbial isolates are reported in TableS1, details on therapeutic regimens in Table S2 and Table S3.

Over a third of the study population (95/224, 42.4%) experienced ≥ 1 IVFOF-related AEs, with therapy discontinuation in 38/224 (17.0%) (Table 1). AEs occurred at a median time of 4.0 (2.0–7.0) days from IVFOF initiation. Hypernatremia was the most frequent AE (53/224, 23.7%), followed by hypokalaemia (22/224 9.8%), diarrhoea (20/224, 8.9%), hypertransaminasemia (12/224, 5.4%), nausea (12/224, 5.4%) and cardiac events (5/224, 2.2%). Patients who developed AEs were older and had a more severe clinical condition compared to no-AEs group, with ICU setting and septic shock at IVFOF initiation in 47/95 (49.5%) vs. 34/129 (26.4%) (*p* < 0.001) and 24/95 (25.5%) vs. 11/129 (8.5%) (*p* < 0.001), respectively. IVFOF was started for lower respiratory tract infections more frequently in AEs group than no-AEs group (62/95 (65.3%) vs. 62/129 (48.1%), p 0.011). No significant differences were observed in IVFOF daily dose.

### TDM subgroup

Among the study population, 68/224 patients (30.4%) underwent IVFOF TDM, with 33/68 (48.5%) having more than one TDM assessment. Patients assigned to TDM had a more severe baseline condition compared to no-TDM patients, with septic shock at IVFOF initiation in 16/68 (23.9%) vs. 19/156 (12.2%) (p 0.028). IVFOF mode of administration differed significantly between groups, with CI in 28/68 (41.2%) of TDM group compared to 6/156 (3.9%) of no-TDM group (*p* < 0.001). No significant differences were observed in IVFOF daily dose, length of treatment nor the occurrence of AEs related to IVFOF. On the contrary, IVFOF dose adjustment not related to AEs occurred more frequently in TDM compared to no-TDM group (17/68 (25.0%) vs. 10/156 (6.4%), *p* < 0.001) (**Table S4**). IVFOF TDM samples were obtained after a median of 3.5 (2.5-6.0) days from therapy initiation. Among the 40 patients treated with IVFOF as II, median Cmin value was 171.5 (68.5-244.5) mg/L. Among the 28 patients treated with IVFOF as CI, median Css value was 188.8 (138.0-329.0) mg/L.

### Factors associated to the development of AEs during treatment

In multivariate analysis, ICU setting (adjusted RR 1.59 (95%CI 1.09–2.31), p 0.016), lower respiratory tract infection (adjusted RR 1.46 (95%CI 1.03–2.07), p 0.031) and septic shock at IVFOF initiation (adjusted RR 1.73 (95%CI 1.27–2.37), *p* < 0.001) resulted associated with the development of AEs. Age, baseline alteration of renal function or the presence of any chronic illness did not increase the risk of AEs. Likewise, IVFOF daily dose nor mode of administration (CI compared to II) were related to AEs during treatment (Table 2).

**Table 2:** Univariate and multivariate analysis of the association of selected demographic, clinical and therapeutic factors to the development of AEs during IVFOF treatment

	No AES (*N* = 129)	AES (*N* = 95)	RR [95% CI]	*p*-value	RR _adjusted_ [95% CI] ^a^	*p*-value
Demographics and anamnestic data						
Age in years	59.0 (46.0–71.0)	66.0 (55.0–73.0)	**1.01** **(1.00-1.02)**	**0.041**	1.01 (0.99–1.02)	0.082
≥ 1 comorbidities ^b^	81 (55.5)	65 (44.5)	1.14 (0.82–1.59)	0.433	0.98 (0.69–1.40)	0.914
**Clinical and laboratory data at IVFOF initiation**						
Lower respiratory tract infection	62 (50.0)	62 (50.0)	**1.52** **(1.09–2.11)**	**0.014**	**1.46** **(1.03–2.07)**	**0.031**
Septic shock with Vasopressors need ^b^	11 (31.4)	24 (68.6)	**1.84** **(1.38–2.46)**	**< 0.001**	**1.73** **(1.27–2.37)**	**< 0.001**
eGFR, ml/min/1.73m^2^ ≤ 30^b, c^	6 (37.5)	10 (62.5)	1.55 (1.02–2.35)	**0.041**	1.30 (0.82–2.05)	0.260
Patient’s setting at IVFOF initiation						
IVFOF started in non-intensive wards, patient never admitted to ICU*	78 (68.4)	36 (31.6)	1^*^		1^*^	
IVFOF started before ICU stay	6 (60.0)	4 (40.0)	1.27 (0.57–2.84)	0.565	1.43 (0.63–3.24)	0.386
IVFOF started in ICU	34 (42.0)	47 (58.0)	**1.84** **(1.32–2.55)**	**< 0.001**	**1.59** **(1.09–2.31)**	**0.016**
IVFOF started after ICU stay	11 (57.9)	8 (42.1)	1.33 (0.74–2.41)	0.341	1.28 (0.68–2.41)	0.448
**Treatment data**						
IVFOF median daily dose, grams	16.0 (12.0–24.0)	18.0 (13.4–24.0)	1.02 (0.99–1.04)	0.156	1.01 (0.99–1.04)	0.409
IVFOF starting mode of administration:						
Intermittent infusion*	116 (61.1)	74 (39.0)	1^*^		1	
Continuous infusion	13 (38.2)	21 (61.8)	**1.59** **(1.15–2.18)**	**0.005**	1.23 (0.88–1.73)	0.220

Data are presented as n (row percentages) according to the development (or not) of at least one AEs. Crude and adjusted RRs their relative 95% CI and p-values are reported

Legend: TDM therapeutic drug monitoring, KDIGO kidney disease improving global outcomes, ICU intensive care unit, eGFR estimated glomerular filtration rate

^*^ Reference category

^a^ RRs estimates adjusted by age (years), presence of septic shock with vasopressors support and mode of IVFOF administration

^b^ Sum does not add to the total because of missing values: 1 for at least one comorbidity, 1 for septic shock with vasopressors need, and 1 for eGFR, ml/min/1.73m2 < = 30 ml/min/1.73m2 at the moment of IVFOF start

^c^*N* = 204, excluding 18 patients in renal replacement therapy at the initiation of IVFOF and 1 patient for whom the information was not available

Within IVFOF-based combination regimens, no specific antibiotic was found associated to the development of AEs (Table S5).

Among the 68 patients who underwent TDM, 37 (54.4%) developed ≥ 1 AEs during IVFOF treatment, with events occurring within 5 days after TDM assessment in 18 patients (12 with IVFOF II and 6 with IVFOF CI) (Supplementary Fig. S1). Patients who developed AEs_≤ 5days_ showed higher TDM levels compared to those who did not, albeit not reaching statistical significance. When comparing patients who did and did not develop AEs_≤ 5days_, Cmin median values were 211.3 (113.0-302.5) mg/L vs. 140.6 (64.9-227.2) mg/L (p 0.167), Css median values were 241.0 (164.5–369.0) mg/L vs. 146.0 (111.0-314.0) mg/L (p 0.269), respectively. Receiver operating characteristic (ROC) curves showed AUC of 0.65 (95%CI 0.44–0.86) for Cmin and AUC of 0.67 (0.39–0.95) for Css. When comparing patients who developed hypernatremia_≤ 5days_ (the most frequent AE registered) to those who did not develop AEs_≤ 5days_, Cmin median values were 419.5 (266.0-655.0) mg/L vs. 140.6 (64.9-227.2) mg/L (p 0.012), Css median values were 294.0 (188.0-369.0) mg/L vs. 146.0 (111.0-314.0) mg/L (p 0.103), respectively. ROC curve showed good discriminatory ability for Cmin with AUC of 0.91 (95%CI 0.79-1.0), and weak discriminatory ability for Css with AUC of 0.76 (0.52-1.0). (Supplementary Figures S1-S3).

## Discussion

Our study provides real world data on the use of IVFOF-based regimens in clinical practice, particularly in treating severe infections caused by MDROs. AEs occurred in more than 40% of the study population and were mainly associated to critically ill conditions at IVFOF initiation, although treatment interruption was needed in only a minority of cases. We also highlighted a possible role of IVFOF TDM in predicting the development of AEs during treatment.

With over 220 patients evaluated, this study finds its place among the largest clinical studies on IVFOF so far. Over the last 20 years, only 4 randomized clinical trials (RCT) were published, with the remaining being prospective or retrospective observational studies. Great heterogeneity exists in published literature on patients’ severity and infection type. ICU population ranged from null to over 90% of examined patients. [4, 8, 33, 34] The majority of studies focused on gram-negative MDROs, but a few evaluated specifically infections by gram-positive bacteria. [35–38] Several types of infections have been reported, with pneumonia [39] (including pulmonary exacerbations in patient with cystic fibrosis [40]), urinary tract infections [5–7] and osteoarticular [36, 37, 41] infections being the most frequent. Consistently with the real-life study performed in 2012 in France by Dinh et al., [42] in our cohort 40% of patients started IVFOF in ICU and 15% had septic shock requiring vasopressors at treatment initiation, with gram-positive bacteria in slightly less than a third of cases.

The rate of AEs during treatment observed in our patients was above  40%, higher than what reported in most observational studies but comparable to findings of RCTs. [5, 6, 38, 43] This is likely due to the application of the same classification criteria for AEs, [29] but is also related to the setting of IVFOF use with 40% of treatment started in the ICU. Of note, in our cohort the factors independently associated with the development of AEs were ICU setting, the presence of deep-seated infections (lower respiratory tract infections) and septic shock at IVFOF initiation.

With 23.7% of the total study population experiencing moderate to severe hypernatremia during treatment and only 3.6% already hypernatremic at IVFOF initiation, our study confirmed the high sodium intake related to the drug, which could constitute an issue especially in patients with pre-existing heart conditions or renal failure. The finding, however, may have limited reliability as it is influenced by possible confounders (concomitant therapies, renal failure, critically-ill conditions) particularly in the ICU population, where hypernatremia is reported in 6–26% of patients. [44]

Overall, our findings support recent reviews corroborating the good safety profile of IVFOF, [9, 45] as AEs were generally non-serious and led to discontinuation of treatment in less than 20% of cases.

TDM-guided therapy of glycopeptides, aminoglycosides and, more recently, betalactams has shown that dose modification based on validated PK/PD targets is associated with better clinical outcomes, in terms of both efficacy and the reduction of AEs and resistance during treatment. [13, 15, 46]

Clinical data on IVFOF TDM are currently limited to animal models, case reports and case series [17–21, 47, 48] but no focus has been placed on its correlation to drug toxicity so far. Cojutti et al. described clinical efficacy of the combination therapy with CI IVFOF plus meropenem in bacteriemic VAP caused by MDR *Klebsiella pneumoniae*, with real-time TDM-based program allowing to achieve optimal PK/PD indexes [18]. Gatti et al. reported the effective treatment of post-neurosurgical ventriculitis caused by carbapenem-resistant-*Pseudomonas aeruginosa* with IVFOF plus ceftazidime/avibactam using TDM-guided approach [19]. The same authors reported positive outcomes in 6 patients with BSI and/or pneumonia caused by DTR (difficult-to-treat resistance)-*Pseudomonas aeruginosa* treated with CI IVFOF plus extended-infusion cefiderocol or continuous-infusion ceftazidime-avibactam, demonstrating that microbiological eradication was associated with the obtainment of pre-specified PK/PD targets. Median IVFOF Css was 504.9 (363.2–647.2) mg/L, higher than what observed in our study [21]. However, no treatment-related AEs was reported in any of the studies above. By contrast, in a German observational study on 17 patients with ventriculitis treated with 24 g/day CI IVFOF, median Css was 200 (159–289) mg/L, comparable with our findings. Cases of hypernatremia were reported, particularly in patients with high sodium levels prior to IVFOF start, but correlation with IVFOF TDM was not studied. [49]

In our cohort, nor IVFOF daily dose nor specific antibiotic combination regimen was associated with the development of AEs. On the other hand, albeit based on very few observations which limited the statistical significance of our results, we found a potential role of TDM in predicting IVFOF-related AEs that occur close to TDM assessment.

Our study has limitations. Firstly, the retrospective design, the lack of a control group and the presence of possible confounders (e.g., high proportion of ICU patients and organ dysfunction, use of combination therapy) warrant some caution in associating AEs to IVFOF alone. Yet, this is the most common real-life scenario where the drug is employed, and all the AEs in analysis are known to be associated with IVFOF. [8, 9, 50] As stated before, the frequency and types of AEs in our cohort were consistent with RCTs and prospective, multicenter studies. [4–6, 38, 43]. Secondly, the relatively small sample size, particularly in the TDM group and in the II and CI subgroups, have likely limited the statistical power of the association between IVFOF TDM and the development of AEs. Moreover, the choice to perform TDM was made on a case-by-case basis by the ID consultant, depending on patients’ severity and risk of microbiological failure. Further studies are needed to confirm TDM role in predicting drug toxicity during IVFOF treatment and to establish and validate cut-off values. Lastly, since MIC values were not available for all the bacterial isolates, we could not calculate PK/PD indexes associated to drug efficacy. This should be further investigated, but was beyond the scope of our current work.

## Conclusions

Our real-life data confirms IVFOF-based combination regimens as promising and feasible options for the treatment of severe infections. In this setting, drug-related AEs are expected in a significant proportion of patients, especially in those with a baseline critical condition, but treatment discontinuation is needed in only a minority of cases. IVFOF TDM worth future research, since it may represent a valid tool not only to achieve effective PK/PD targets but also to reduce drug-related AEs.

### Electronic supplementary material

Below is the link to the electronic supplementary material.

Supplementary Material 1

## Data Availability

Anonymized data were abstracted on standardized data collection forms in the web platform REDCap (Reaserch Electronic Data Capture).

## Abbreviations

AE: Adverse Events
AUROC: Area Under the Receiver Operating Characteristic
AUC: Area Under the Curve
CI: Continuous Infusion
Cmin: Minimum Concentration
CRRT: Continuous Renal Replacement Therapy
Css: Steady-State Concentration
CTCAE: Common Terminology Criteria for Adverse Events
DTR: Difficult-to-Treat Resistance
ECOFF: Epidemiologic Cutoff
eGFR: Estimated Glomerular Filtration Rate
ESBL: Extended Spectrum Beta-Lactamases
ICU: Intensive Care Unit
II: Intermittent Infusion
IVFOF: Intravenous Fosfomycin
KDIGO: Kidney Disease Improving Global Outcomes
MDROs: Multidrug-Resistant Organisms
MIC: Minimal Inhibitory Concentration
PK/PD: Pharmacokinetic/Pharmacodinamic
RCT: Randomized Clinical Trials
TDM: Therapeutic Drug Monitoring
VAP: Ventilator Associated Pneumonia
