# Supplementary material for: Adverse events during intravenous fosfomycin therapy in a real-life scenario. Risk factors and the potential role of therapeutic drug monitoring
Source: BMC Infect Dis. 2024 Jun 28;24:650. doi: 10.1186/s12879-024-09541-4 (PMC11212171; doi:10.1186/s12879-024-09541-4)
Supplement: Supplementary file 1 — Supplementary Material 1 [file 12879_2024_9541_MOESM1_ESM.docx]

**Adverse Events during intravenous fosfomycin therapy in a real-life scenario. Risk factors and the role of therapeutic drug monitoring**

**SUPPLEMENTARY MATERIAL**

**Table S1** Microbial isolates identified among the 203 documented bacterial infections treated with IVFOF-based regimens

**Table S2** Antibiotics used in combination with IVFOF for gram-negative bacteria: *Enterobacterales* (including ESBL producers and CRE), *Pseudomonas aeruginosa* (including DTR Pseudomonas)

**Table S3** Antibiotics used in combination with IVFOF for gram-positive bacteria: *Staphylococcus aureus* (including MRSA), *Enterococcus* spp (including VRE)

**Table S4** Clinical characteristics of the study population. Comparison between therapeutic drug monitoring (TDM) group and no-TDM group

**Table S5** Association between concomitant antibiotics and the development of AEs during IVFOF therapy

**Figure S1** Association of IVFOF TDM values with the development of AEs. For evaluating the predictive value of TDM, only patients with AEs occurred within 5 days after TDM assessment were retained in the analysis

**Figure S2** Association of IVFOF TDM values with the development of AEs in patients treated with IVFOF intermittent infusion (n=36**). Panel A)** ROC curve of Cmin for the development of at least 1 AEs within 5 days after TDM assessment (n=12). **Panel B)** ROC curve of Cmin for the development of hypernatremia within 5 days after TDM assessment (n=4)

**Figure S3** Association of IVFOF TDM values with the development of AEs in patients treated with IVFOF continuous infusion (n=22). **Panel A)** ROC curve of Css for the development of at least 1 AEs within 5 days after TDM assessment (n=6). **Panel B)** ROC curve of Css for the development of hypernatremia within 5 days after TDM assessment (n=5)

**Table S1**. Microbial isolates identified among the 203 documented bacterial infections treated with IVFOF-based regimens

|  | Overall  N=257 |
| --- | --- |
| GRAM-NEGATIVE BACTERIA | 178 (69.3) |
|  |  |
| *Enterobacterales* | 74 (28.8) |
| - *Enterobacter spp^a^* | 10 (3.9) |
| - *E. coli* | 21 (8.2) |
| - *Klebsiella pneumoniae* | 32 (12.5) |
| - *Klebsiella oxytoca* | 4 (1.6) |
| - *Proteus spp* | 3 (1.2) |
| - *Serratia spp* | 3 (1.2) |
| - *Morganella morganii* | 1 (0.4) |
| - ESBL-E*nterobacterales ^b^* | 7 (2.7) |
| - Carbapenem-Resistant E*nterobacterales ^c^* | 15 (5.8) |
| *Pseudomonas aeruginosa* | 99 (38.5) |
| - MDR *Pseudomonas aeruginosa* | 53 (20.5) |
| - DTR *Pseudomonas aeruginosa* | 21 (8.2) |
| *Nonfermenting gram-negative. other than P. aeruginosa ^d^* | 5 (1.9) |
|  |  |
| GRAM-POSITIVE BACTERIA | 79 (30.7) |
|  |  |
| *Staphylococcus aureus* | 63 (24.5) |
| - Methicillin-resistant *Staphylococcus aureus* | 20 (7.8) |
| *Enterococcus faecalis* | 5 (1.9) |
| *Enterococcus faecium* | 6 (2.3) |
| - Vancomycin-resistant *Enterococcus faecium* | 4 (1.6) |
| Coagulase-negative *Staphylococc*i | 4 (1.6) |
| *Streptococcus spp.* | 1 (0.4) |

Legend: *ESBL extended-spectrum beta-lactamase, MDR multidrug resistant DTR difficult-to-treat resistant*

*^a^ Enterobacter spp: 4/10 Enterobacter aerogenes, 4/10 Enterobacter hormaechei, 2/10 Enterobacter cloacae*

*^b^ ESBL****:*** *3/7 Enterobacter spp, 2/7 Klebsiella pneumoniae, 2/7 E.coli.*

*^c^ CRE: 15/15 Klebsiella pneumoniae carbapenemase KPC- producing Enterobacterales; among them 14/15 Klebsiella pneumoniae, 1/15 E.coli.*

*^d^ Nonfermenting gram-negative other than P. aeruginosa: 3/5 Stenotrophomonas maltophilia, 1/5 Burkholderia cenocepacia Genomovar III, 1/5 Achromobacter xylosoxidans.*

***Table S2*** *Antibiotics used in combination with IVFOF for gram-negative bacteria: Enterobacterales (including ESBL producers and CRE), Pseudomonas aeruginosa (including DTR Pseudomonas)*

| **Combination therapy with IVFOF** | **Enterobacterales (N=74)**  **n,%** | **ESBL-E (N=7) n,%** | **CRE (N=15) n,%** | **P.aeruginosa (N=99)**  **n,%** | **DTR P.aeruginosa (N=21)**  **n,%** |
| --- | --- | --- | --- | --- | --- |
| Monotherapy | 3 (4.1) | - | - | - | - |
| Third/fourth generation cephalosporins | 24 (32.4) | 1 (14.3) | - | 30 (30.3) | 3 (14.3) |
| Piperacillin/tazobactam | 13 (17.6) | 1 (14.3) | - | 9 (9.1) | 2 (9.5) |
| Carbapenems | 16 (21.6) | 4 (57.1) | 2 (13.3) | 29 (29.3) **^b^** | 7 (33.3)**^b^** |
| New agents (BL/BLI and cefiderocol) | 17 (23.0) ^a^ | - | 13 (86.7) | 28 (28.3) ^c^ | 9 (42.9) ^c^ |
| Aminoglycosides | 1 (1.4) | 1 (14.3) | - | - | - |
| Fluoroquinolones | - | - | - | 3 (3.0) ^d^ | - |

Legend: BL/BLI: beta-lactam/beta-lactamases inhibitors, ESBL: extended-spectrum beta-lactamase, CRE: carbapenems resistant enterobacterales, IVFOF intravenous fosfomycin, DTR: difficult to treat

^a^ 1/17 Cefiderocol (K.pneumoniae), 8/17 Ceftazidime/avibactam (K.pneumoniae), 4/17 Ceftolozane/tazobactam (2 K.pneumoniae 1 E.coli 1 Serratia due to coinfection with P.aeruginosa), 3/17 Meropenem/vaborbactam (K.pneumoniae), 1/17 Colistin+ Ceftazidime/avibactam (K.pneumoniae)

**^b^** 7/16 (4/6 DTR) Meropenem + Colistin due to ceftolozane/tazobactam shortage notified on 23 December 2020 (https://www.aifa.gov.it/en/-/nota-informativa-importante-su-zerbaxa)

^c^ 11/28 Ceftazidime/avibactam, 15/28 Ceftolozane/tazobactam, 1/28 Cefiderocol, 1/28 Ceftolozane/tazobactam + Colistin

^d^ 1/3 Fluoroquinolone + Colistin

**Table S3** Antibiotics used in combination with IVFOF for gram-positive bacteria: Staphylococcus aureus (including MRSA), Enterococcus spp (including VRE)

| **Combination therapy with IVFOF** | **S.aureus, (N=63) n,%** | **MRSA (N=20) n,%** | **E.faecalis (N=5)**  **n,%** | **E.faecium (N=6)**  **n,%** | **VRE**  **(N=4)**  **n,%** |
| --- | --- | --- | --- | --- | --- |
| Oxacillin/Cefazoline | 26 (41.3) | - | - | - | - |
| Daptomycin | 10 (15.9) | 5 (25.0) | 1 (20.0) | 2 (33.3) | 1 (25.0) |
| Vancomycin | 7 (11.1) | 7 (35.0) | - | - | - |
| Ceftaroline | 2 (3.2) | 2 (10.0) | - | - | - |
| Ampicillin | - | - | 3 (60.0) | - | - |
| Linezolid | 3 (4.8) | 3 (15.0) | - | 2 (33.3) | 2 (50.0) |
| Daptomycin + Ceftaroline | 4 (6.4) | 3 (15.0) | - | - | - |
| Daptomycin+Tigecycline | - | - | - | 2 (33.3) | 1 (25.0) |
| Daptomycin+Ampicillin | - | - | 1 (20.0) | - | - |
| Trimetoprim/Sulfamethoxazole | 1 (1.6) | - | - | - | - |
| Other beta-lactams with activity against MSSA^a^ | 10 (15.9) | - | - | - | - |

Legend: IVFOF intravenous fosfomycin, MSSA Methicillin susceptible S.aureus, MRSA Methicillin-resistant S.aureus, VRE Vancomycin-resistant Enterococcus

^a^ due to coinfection with gram-negative bacteria

***Table S4*** *Clinical characteristics of the study population. Comparison between therapeutic drug monitoring (TDM) group and no-TDM group*

|  | **no-TDM**  **(N=156)** | **TDM**  **(N=68)** | **p-value** |
| --- | --- | --- | --- |
| **Demographics** |  |  |  |
| Age, years | 62.0 (46.0-71.0) | 65.5 (53.5-73.0) | 0.220 |
| Gender, female | 61 (39.1) | 25 (36.8) | 0.741 |
| Ethnicity, Caucasian | 137 (87.8) | 64 (94.1) | 0.230 |
| Comorbidities |  |  |  |
| - At least 1 comorbidity ^a^ | 97 (62.6) | 49 (72.1) | 0.171 |
| - Myocardial infarction | 16 (10.3) | 9 (13.2) | 0.515 |
| - Chronic pulmonary disease | 30 (19.2) | 9 (13.2) | 0.277 |
| - Mild or severe liver disease | 14 (9.0) | 10 (14.7) | 0.202 |
| - Diabetes Mellitus | 43 (27.6) | 24 (35.3) | 0.245 |
| Charlson Comorbidity Index |  |  |  |
| - 0 | 58 (37.2) | 19 (27.9) | 0.306 |
| - 1 | 32 (20.5) | 16 (23.5) |  |
| - 2 | 31 (19.9) | 12 (17.7) |  |
| - 3 | 23 (14.7) | 10 (14.7) |  |
| - ≥4 | 12 (7.7) | 11 (16.2) |  |
| **Clinical and laboratory data at IVFOF initiation** |  |  |  |
| Patient’s ward |  |  |  |
| - ICU ^b^ | 56 (35.9) | 25 (36.8) | 0.901 |
| - Non-intensive wards | 100 (64.1) | 43 (63.2) |  |
| Infection site ^c^ |  |  |  |
| - BSI | 44 (28.2) | 28 (41.2) | 0.056 |
| - Primary BSI | 14 (9.0) | 5 (7.4) | 0.799 |
| - Lower respiratory tract infection | 90 (57.7) | 34 (50.0) | 0.287 |
| - VAP | 51 (32.7) | 22 (32.4) | 0.960 |
| - Surgical site infection | 7 (4.5) | 5 (7.4) | 0.519 |
| - Urinary tract infection | 12 (7.7) | 1 (1.5) | 0.116 |
| - Skin and soft tissue infection | 7 (4.5) | 4 (5.9) | 0.739 |
| - Cardiovascular infection | 9 (5.8) | 8 (11.8) | 0.119 |
| - Osteoarticular infection | 9 (5.8) | 7 (10.3) | 0.227 |
| - Others | 11 (7.1) | 12 (17.7) | **0.016** |
| Septic shock with vasopressors need ^a^ | 19 (12.2) | 16 (23.9) | **0.028** |
| eGFR, ml/min/1.73m^2^ ^a,d^ | 93.9 (56.6-112.3) | 90.9 (70.8-106.8) | 0.587 |
| eGFR, ml/min/1.73m^2^ <= 30 ^a,d^ | 12 (8.2) | 4 (6.9) | 1.000 |
| Hypernatremia | 5 (3.2) | 3 (4.4) | 0.701 |
| **Microbiological data at IVFOF initiation** |  |  |  |
| Non-identified pathogen | 20 (12.8) | 1 (1.5) | **0.005** |
| Identified pathogen | 136 (87.2) | 67 (98.5) |  |
| - Monomicrobial infection ^e^ | 104 (76.5) | 49 (73.1) | 0.604 |
| - Polymicrobial infection ^e^ | 32 (23.5) | 18 (26.9) |  |
| Infection sustained by MDROs ^e^ | 67 (49.3) | 32 (47.8) | 0.840 |
| **Treatment data & AEs** |  |  |  |
| IVFOF daily dose, grams | 18.0 (12.0-24.0) | 16.0 (13.2-23.4) | 0.196 |
| Days elapsed from pathogens identification to IVFOF start | 3.0 (1.0-7.0) | 2.5 (1.0-5.0) | 0.206 |
| IVFOF mode of administration |  |  |  |
| - Intermittent | 150 (96.2) | 40 (58.8) | **<0.001** |
| - Continuous infusion | 6 (3.9) | 28 (41.2) |  |
| Length of IVFOF therapy, days | 10.0 (6.0-16.0) | 13.0 (8.0-18.0) | **0.015** |
| Dosage adjustment of IVFOF (increasing/decreasing dose) not related to AEs | 10 (6.4) | 17 (25.0) | **<0.001** |
| AEs, related to IVFOF |  |  |  |
| - ≥ 1 AE | 58 (37.2) | 37 (54.4) | **0.016** |
| - Diarrhoea | 12 (7.7) | 8 (11.8) | 0.326 |
| - Nausea | 7 (4.5) | 5 (7.4) | 0.519 |
| - Hypernatremia | 30 (19.2) | 23 (33.8) | **0.018** |
| - Hypertransaminasemia | 8 (5.1) | 4 (5.9) | 0.758 |
| - Hypokalemia | 13 (8.3) | 9 (13.2) | 0.257 |
| - Cardiac events ^f^ | 5 (3.2) | 0 (0.0) | 0.326 |
| Days elapsed from IVFOF start to first AE | 4.0 (3.0-7.0) | 4.0 (2.0-8.0) | 0.620 |

Legend: AE Adverse event, BSI blood stream infection, CTCAE common terminology criteria for adverse events, eGFR estimated glomerular filtration rate, ICU intensive care unit, IVFOF intravenous fosfomycin, KDIGO kidney disease improving global outcomes, MDROs multidrug resistant organisms, TDM therapeutic drug monitoring, VAP ventilator associated pneumonia;

^a^ Sum does not add to the total because of missing values: 1 for at least one comorbidity, 1 for Septic shock with vasopressors need, and 1 for eGFR, ml/min/1.73m2 <= 30

^b^ 5/156 (3.2%) patients in the no-TDM group and 5/68 (7.4%) patients in the TDM group started IVFOF before ICU admission. 14/156 (9.0%) patients in the no-TDM group and 5/68 (7.4%) patient in the TDM group started IVFOF after ICU stay.

^c^ 63 patients (28%) had multiple site infections. 33/156 (21.2%) were in the no-TDM group and 30/68 (44.1%) were in the TDM group. Further details available in supplementary Table S2.

^d^ N= 204, excluding the 18 patients (9 in the no-TDM group an 9 in the TDM group) in dialysis/CCRT and 1 TDM patient for whom the information about dialysis was not available;

^e^ N= 203, excluding the 21 patients for whom pathogen was not identified;

^f^ Cardiac events defined as development of arrhythmias, QT prolongation, cardiac arrest.

**Table S5** Association between concomitant antibiotics and the development of AEs during IVFOF therapy

|  | **Overall** | **No-AE**  **(N=129)** | **AE**  **(N=95)** | **p-value** |
| --- | --- | --- | --- | --- |
| Number of concomitant antibiotics |  |  |  |  |
| IVFOF only | 4 (1.8) | 2 (1.6) | 2 (2.1) | 0.860 |
| 1 | 116 (51.8) | 69 (53.5) | 47 (49.5) |  |
| 2 | 75 (33.5) | 43 (33.3) | 32 (33.7) |  |
| ≥3 | 29 (13.0) | 15 (11.6) | 14 (14.7) |  |
| Type of concomitant antibiotics* |  |  |  |  |
| Third/fourth generation cephalosporins |  |  |  |  |
| No | 167 (74.6) | 99 (76.7) | 68 (71.6) | 0.476 |
| Yes | 48 (21.4) | 24 (18.6) | 24 (25.3) |  |
| Ongoing but not part of IVFOF-based regimen | 9 (4.0) | 6 (4.7) | 3 (3.2) |  |
| Piperacillin/tazobactam |  |  |  |  |
| No | 189 (84.4) | 112 (86.8) | 77 (81.1) | 0.508 |
| Yes | 26 (11.6) | 13 (10.1) | 13 (13.7) |  |
| Ongoing but not part of IVFOF-based regimen | 9 (4.0) | 4 (3.1) | 5 (5.3) |  |
| Carbapenems |  |  |  |  |
| No | 167 (74.6) | 100 (77.5) | 67 (70.5) | 0.172 |
| Yes | 47 (21.0) | 26 (20.2) | 21 (22.1) |  |
| Ongoing but not part of IVFOF-based regimen | 10 (4.5) | 3 (2.3) | 7 (7.4) |  |
| New agents (BL/BLI and cefiderocol) |  |  |  |  |
| No | 183 (81.7) | 108 (83.7) | 75 (79.0) | 0.298 |
| Yes | 39 (17.4) | 19 (14.7) | 20 (21.1) |  |
| Ongoing but not part of IVFOF-based regimen | 2 (0.9) | 1 (1.6) | 0 (0.0) |  |
| Oxacillin or Cefazoline |  |  |  |  |
| No | 197 (88.0) | 113 (87.6) | 84 (88.4) | 0.852 |
| Yes | 27 (12.0) | 16 (12.4) | 11 (11.6) |  |
| Ongoing but not part of IVFOF-based regimen | - | - | - |  |
| Daptomycin |  |  |  |  |
| No | 185 (82.6) | 102 (79.1) | 83 (87.4) | 0.204 |
| Yes | 26 (11.6) | 19 (14.7) | 7 (7.4) |  |
| Ongoing but not part of IVFOF-based regimen | 13 (5.8) | 8 (6.2) | 5 (5.3) |  |
| Vancomycin |  |  |  |  |
| No | 208 (92.9) | 121 (93.8) | 87 (91.6) | 0.838 |
| Yes | 11 (4.9) | 5 (3.9) | 6 (6.3) |  |
| Ongoing but not part of IVFOF-based regimen | 5 (2.2) | 3 (2.3) | 2 (2.1) |  |
| Linezolid |  |  |  |  |
| No | 206 (92.0) | 119 (92.3) | 87 (91.6) | 0.551 |
| Yes | 7 (3.1) | 5 (3.9) | 2 (2.1) |  |
| Ongoing but not part of IVFOF-based regimen | 11 (4.9) | 5 (3.9) | 6 (6.3) |  |

Legend: TDM therapeutic drug monitoring, BL/BLI beta-lactam/beta-lactamases inhibitors;

* all antibiotics administered in concomitance with IVFOF are reported. When administered for other pathogens but were not part of the IVFOF-based regimen, are specified as “ongoing but not part of IVFOF-based regimen”

**Figure S1** Association of IVFOF TDM values with the development of AEs. For evaluating the predictive value of TDM, only patients with AEs occurred within 5 days after TDM assessment were retained in the analysis


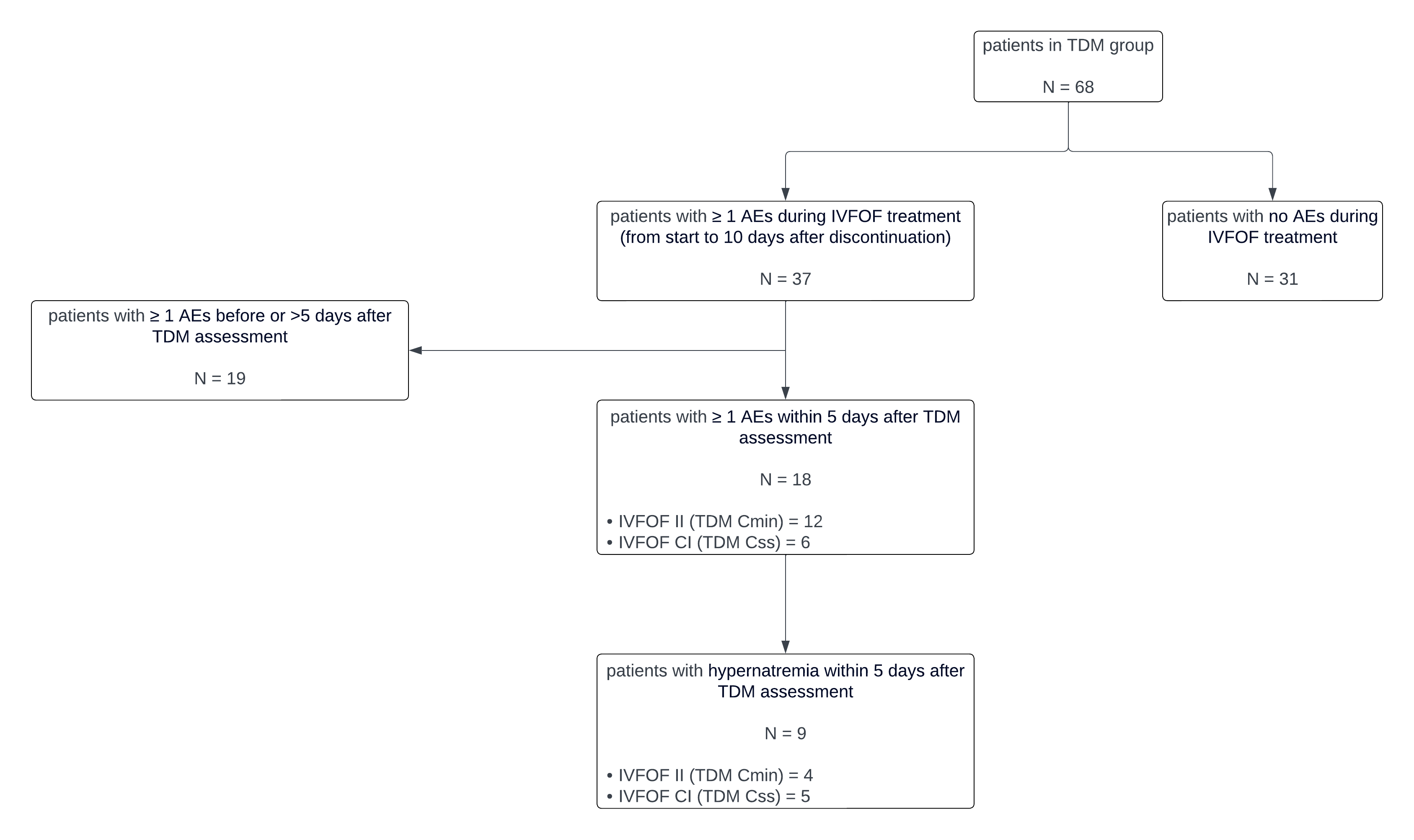


|  | AEs  N = 37 | no-AEs  N = 31 | p value |
| --- | --- | --- | --- |
| Length of IVFOF therapy, days | 12 (8 – 16) | 16 (10 – 28) | 0.042 |
| Time from IVFOF start to TDM assessment, days | 3 (2- 5) | 4 (3- 8) | 0.202 |

**Figure S2** Association of IVFOF TDM values with the development of AEs in patients treated with IVFOF intermittent infusion (n=36). Panel A) ROC curve of Cmin for the development of at least 1 AEs within 5 days after TDM assessment (n=12). Panel B) ROC curve of Cmin for the development of hypernatremia within 5 days after TDM assessment (n=4)

A


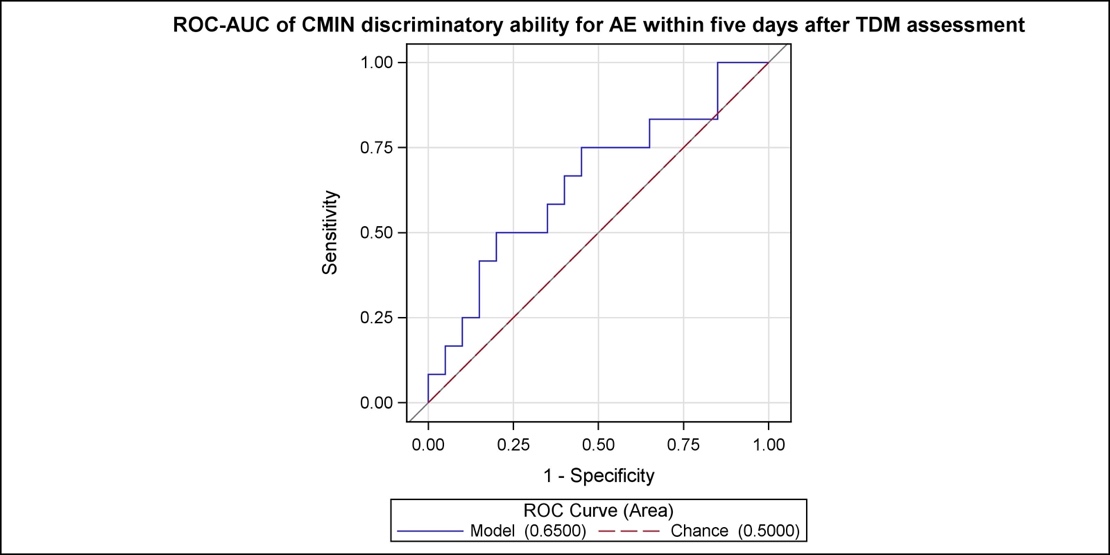


| **AUC** | **95% Wald Confidence Limits** | | **Pr > ChiSq** |
| --- | --- | --- | --- |
| 0.6500 | 0.4438 | 0.8562 | 0.1539 |


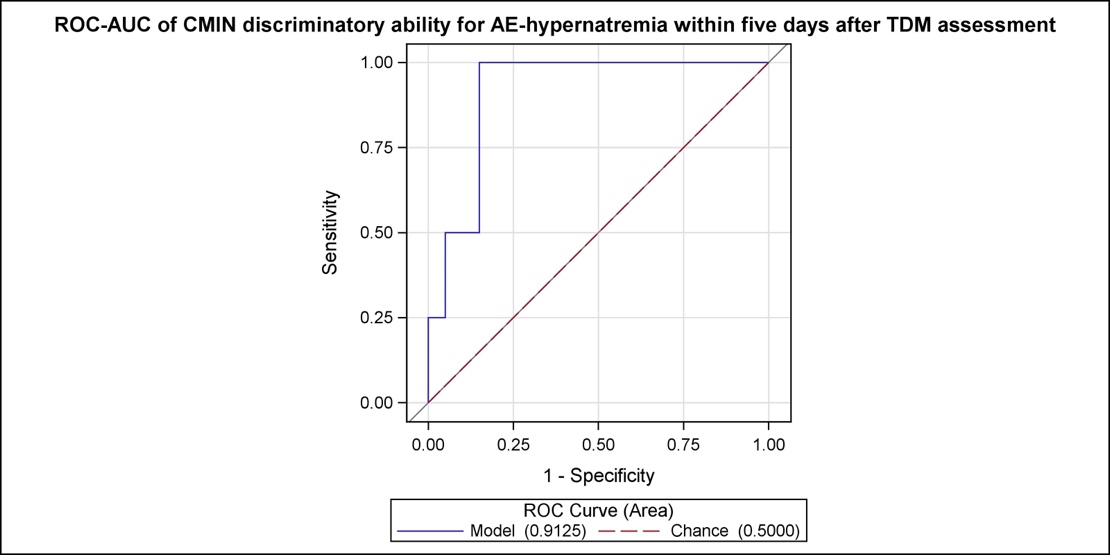
B

| **Area** | **95% Wald Confidence Limits** | | **Pr > ChiSq** |
| --- | --- | --- | --- |
| 0.9125 | 0.7917 | 1.0000 | <.0001 |

**Figure S3** Association of IVFOF TDM values with the development of AEs in patients treated with IVFOF continuous infusion (n=22). Panel A) ROC curve of Css for the development of at least 1 AEs within 5 days after TDM assessment (n=6). Panel B) ROC curve of Css for the development of hypernatremia within 5 days after TDM assessment (n=5)

A


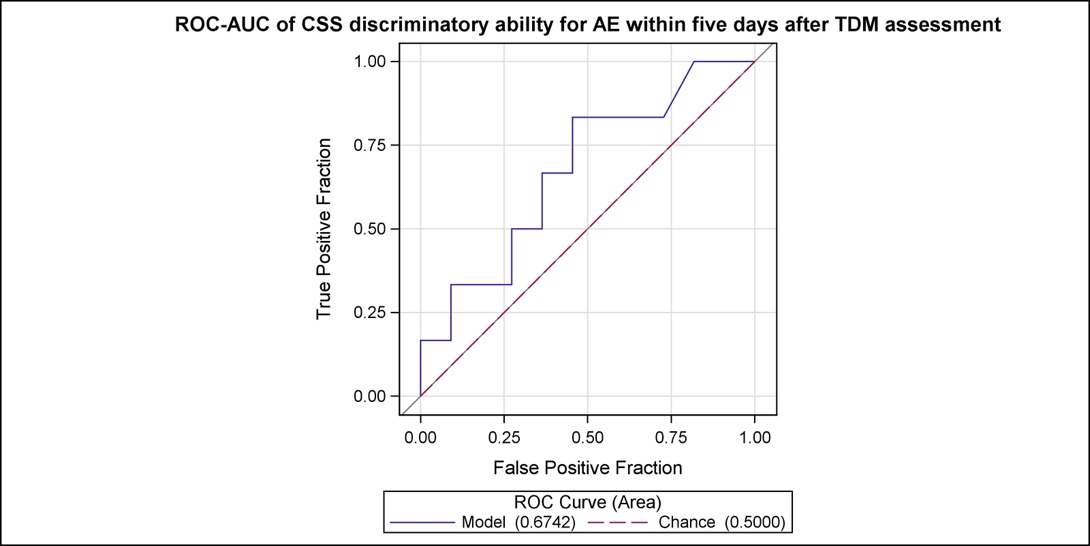


| **Area** | **95% Wald Confidence Limits** | | **Pr > ChiSq** |
| --- | --- | --- | --- |
| 0.6742 | 0.3928 | 0.9557 | 0.2249 |

B


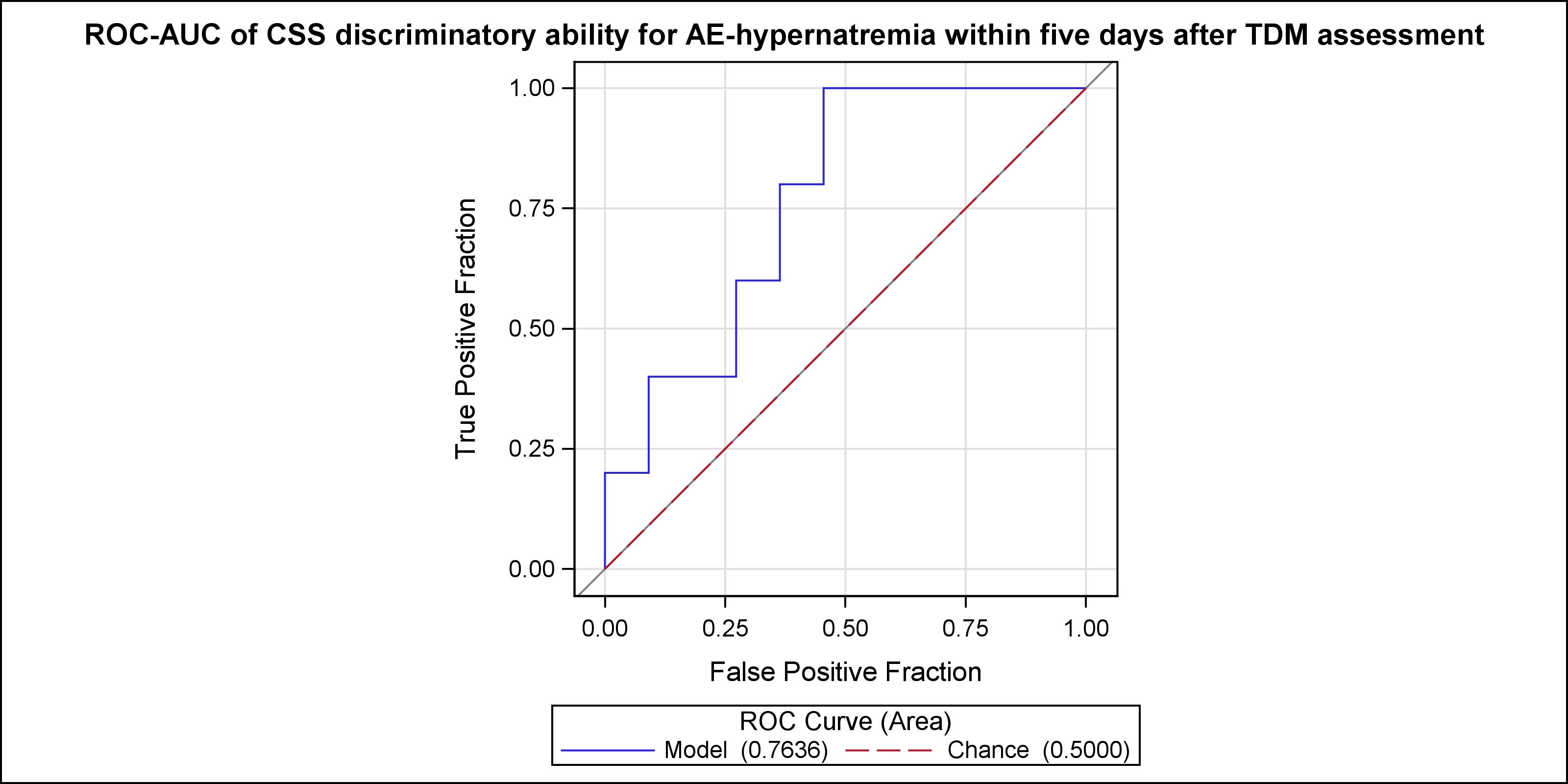


| **Area** | **95% Wald Confidence Limits** | | **Pr > ChiSq** |
| --- | --- | --- | --- |
| 0.7636 | 0.5180 | 1.0000 | 0.0354 |
